# Native llama Nanobody Library Panning Performed by Phage and Yeast Display Provides Binders Suitable for C-Reactive Protein Detection

**DOI:** 10.3390/bios11120496

**Published:** 2021-12-03

**Authors:** Sandra Oloketuyi, Robert Bernedo, Andreas Christmann, Justyna Borkowska, Giulia Cazzaniga, Horst Wilhelm Schuchmann, Joanna Niedziółka-Jönsson, Katarzyna Szot-Karpińska, Harald Kolmar, Ario de Marco

**Affiliations:** 1 Laboratory for Environmental and Life Sciences, University of Nova Gorica, 5000 Nova Gorica, Slovenia; sandra.oloketuyi@ung.si (S.O.); robert.navarro@vib-ugent.be (R.B.); giulia.cazzaniga@unimi.it (G.C.); 2 Applied Biochemistry, Technical University of Darmstadt, 64200 Darmstadt, Germany; Christmann@Biochemie-TUD.de (A.C.); schuchmann@biochemie-tud.de (H.W.S.); Kolmar@Biochemie-TUD.de (H.K.); 3 Institute of Physical Chemistry, Polish Academy of Sciences, 01-224 Warsaw, Poland; justyna150397@gmail.com (J.B.); jniedziolka@ichf.edu.pl (J.N.-J.); kszot@ichf.edu.pl (K.S.-K.)

**Keywords:** phage display, yeast display, nanobodies, biopanning, CRP

## Abstract

C-reactive protein (CRP) is an inflammation biomarker that should be quantified accurately during infections and healing processes. Nanobodies are good candidates to replace conventional antibodies in immunodiagnostics due to their inexpensive production, simple engineering, and the possibility to obtain higher binder density on capture surfaces. Starting from the same pre-immune library, we compared the selection output resulting from two independent panning strategies, one exclusively exploiting the phage display and another in which a first round of phage display was followed by a second round of yeast display. There was a partial output convergence between the two methods, since two clones were identified using both panning protocols but the first provided several further different sequences, whereas the second favored the recovery of many copies of few clones. The isolated anti-CRP nanobodies had affinity in the low nanomolar range and were suitable for ELISA and immunoprecipitation. One of them was fused to SpyTag and exploited in combination with SpyCatcher as the immunocapture element to quantify CRP using electrochemical impedance spectroscopy. The sensitivity of the biosensor was calculated as low as 0.21 μg/mL.

## 1. Introduction

C-reactive protein (CRP) is a plasma protein that participates in the systemic response to inflammation and the synthesis of which rapidly increases within hours of an infection or tissue injuries and apoptosis [1,2]. It can bind to molecular structures exposed on the (bacterial) pathogen surface and present in apoptotic cells that contribute to pathogen clearance by favoring phagocytosis. Its concentration is also used to monitor the inflammatory response in chronic diseases and atherosclerosis. Recent reports indicate that CRP content variation could represent a complementary biomarker to evaluate microvascular risk in patients with type 2 diabetes, the prognosis of pancreatic neoplasm and to follow the progression of a COVID-19 infection [3,4,5]. The involvement of such protein in several physiological disorders has urged the development of diagnostic tools for its quantification. However, the sensitivity and reliability of the available methods differ significantly [6], and there is a need for both new reagents and diagnostic approaches. Generally, IgG antibodies have been used for CRP recognition [7]. However, due to their expansive production process and heterogeneity after functionalization, alternative immunocapture reagents have been proposed as peptides, aptamers and bacteriophages [8,9,10].

In this perspective, we exploited our experience in biopanning for recovering new nanobodies (VHHs) specific for CRP and engineering them into immunocapture diagnostic reagents [11,12,13]. Specifically, we adopted two selection protocols for their isolation, starting from a master library obtained from non-immunized llamas, and compared their output. We initially used a phage display naïve library [14] and performed only two selection rounds to preserve high clone variability and maximize the opportunity to identify nanobodies with different binding characteristics. This procedure was preferred since, similarly to what has been described for the panning of peptide phage libraries, we have previously observed that iterative panning often promoted binders providing very fast replication rather than strong binders [15]. Despite being straight-forward and rapid, the major bottleneck of this protocol is represented by the screening step of the clones recovered from panning to validate their specificity for the antigen. An average panning output yields 10^6^–10^7^ clones and, theoretically, these should be all tested. Since the phage dimension does not enable the *en masse* evaluation of the whole population by flow cytometry and selective sorting, clones are screened individually, usually by low-throughput ELISA. In most of the academic labs, this approach enables the analysis of only a few hundred clones, while labs equipped with robots or high-throughput SPR systems cannot screen more than a few thousand candidates, which still represents a minimal part of the total population of enriched clones. Under these conditions, it can be expected that several sequences corresponding to rare but potentially interesting binders remain undetected after screening. The shortcoming represented by the screening step of binders displayed on phages evidences the comparative advantage that can have yeast or bacterial libraries of recombinant antibodies. In this last case, the whole population of clones obtained after panning can be analyzed using flow cytometry and effective binders selectively sorted. In return, the drawback of such libraries is their lower dimension, and, consequently, their theoretical lower diversity, with respect to phage display libraries. The two technologies have been compared [16] and combined, such as in the case in which peptides displayed on phages have been screened using antigens displayed on yeast and vice versa [17,18,19]. To our knowledge, however, there is no report in which they have been integrated within a combined process to exploit their specific advantages, namely, the larger dimension of phage libraries and the flow-cytometric evaluation of the recovered clones allowed by the yeast format. Therefore, we designed the following protocol: (i) first round of phage display panning starting from the master nanobody library to obtain the round one (R1) phage sub-library; (ii) recovery of the DNA corresponding to the R1 phage sub-library; (iii) DNA recombination to create an R1 yeast display sub-library; (iv) panning of the R1 yeast display sub-library followed by flow cytometry analysis with fluorescent CRP and sorting of the most promising candidates (R2 yeast sub-library); v) comparison of such candidates with those (R2 phage sub-library) recovered by means of a conventional phage display panning and ELISA screening of the R1 sub-library. Finally, the isolated nanobodies were characterized for their biochemical and biophysical features.

## 2. Materials and Methods

### 2.1. Phage Display Nanobody Library Panning

Commercially available CRP from human pleural fluid was purchased from LEE Biosolutions (Maryland Heights, USA) and an aliquot of 10 μg of CRP was buffer exchanged in PBS (pH 8.0) before being used to coat 50 µL of beads (M-450 epoxy magnetic beads, Dynabeads, ThermoFischer Scientific, Waltham, MA, USA), according to the manufacturer’s instructions. An aliquot of 10^12^ phages from a pre-immune VHH phage display library [14] was incubated with 50 µL of epoxy beads in the presence of 1 mL of PBS, 0.1% Tween20 and 2% skimmed milk for 30 min at room temperature. The phage unbound fraction was recovered and underwent another depletion cycle for 90 min at 4 °C in 1.5 mL of the same buffer. The beads were discharged, the unbound phages were incubated for 2 h at 4°C in 1x PBS, 0.1% Tween20, and 2% skimmed milk in the presence of the CRP saturated beads. The beads were washed 20 times with 1 mL of cold PBS plus 0.1% Tween20 and the beads were recovered by using a magnet. Finally, phages were eluted in 1 mL of 0.2 M glycine, pH 2.0, immediately neutralized with 100 µL of 1 M Tris-HCl, pH 9.1. Four hundred microliter of 1 M Tris-HCl, pH 7.4, was added to buffer the sample. TG1 E. coli was grown at 37 °C in minimal medium until OD600 reached the value of 1.0 and 500 µL was used to inoculate 50 mL of 2xTY, 2% glucose medium. When the OD600 of the culture reached 0.5, 750 µL of the selected phages was added to 10 mL of TG1 culture. The infection was induced for 30 min at 37 °C before collecting the bacteria by centrifugation (10 min at 3.100xg), the pellet was diluted in 1.8 mL of 2xTY medium and spread on large Petri dishes prepared with 2xTY medium plus ampicillin. The dishes were incubated overnight at 37 °C, scrubbed, and the bacteria concentration was calculated according to the OD600 value before starting their amplification and a new panning cycle. The total phagemid DNA from the 2nd panning round was isolated using a miniprep kit (Macherey Nagel) and used to transform competent TG1. Single clones were used to infect a deep 96-well plate filled with 2xTY, 2% glucose and ampicillin (100 µg/mL). The plate was incubated overnight at 37 °C and stirred at 240 rpm. The day after, a master plate was made by diluting the single clone cultures in 2xTY, 30% glycerol, ampicillin (100 µg/mL), and stored at −80 °C.

### 2.2. Phage Screening by ELISA

The expression and secretion of nanobody-displaying phages was obtained by growing the transformed bacteria at 37 °C and 250rpm for 2–3 h until OD600 reached the value of 0.5–0.6, then the culture was infected with 10^12^ pfu of M13K07 helper phages. After a 30-min incubation at room temperature without shaking, bacteria were centrifuged for 10 min at 2000 rpm, the supernatant was removed and pellets were resuspended in 2xTY, ampicillin (100 µg/mL) and kanamycin (50 µg/mL). The culture was incubated overnight at 30 °C and the supernatant was used for ELISA using 96-well flat bottom MaxiSorp plates (Nunc). Wells were coated overnight at 4 °C with 0.25 μg/well of CRP before saturation in the presence of 3% BSA-PBS and incubation for 2 h at room temperature with phage samples (100 µL). Controls were performed in the presence of 2% BSA-PBS. Secondary mouse anti-M13 HRP antibodies (GE Healthcare) were incubated for 1 h at room temperature before visualizing the specific signal in the presence of a 3,3′,5,5′-Tetramethylbenzidine (TMB) solution (Sigma, T3405) and reading the absorbance at 450nm. Clones were considered positive when their signal over the background was higher than 5. The DNA of the corresponding clones was recovered for sequencing; unique sequences were identified after alignment. Sequence multiple alignment analysis was performed with Clustal Omega (https://www.ebi.ac.uk/Tools/msa/clustalo/, accessed 21 November 2020) and the basic biophysic features of nanobodies were predicted by ProtParam (https://web.expasy.org/cgi-bin/protparam/protparam, accessed 23 November 2020). 

### 2.3. Yeast Display Panning and Output Evaluation

The DNA recovered from the colonies obtained at the end of the first round of phage display selection, which was used by PCR to amplify the nanobody coding sequences by using the primers VHH bib GG up: ggtggtggtggttctggtggtggtggttctATATATGGTCTCAaacagCCGGCCATGG and VHH bib GG lo: TTACAAGTCCTCTTCAGAAATAAGCTTTTGCTCGGTCTCTGAGCCGGATCCTGAGGAGACGGTGACC.

Yeast library generation was performed as described previously [20]. Briefly, genes were purified and introduced by gap repair cloning into NdeI/BamHI linearized yeast display vector PCT using yeast strain EBY100 [21]. The library generation was performed according to Benatuil et al. [22]. Yeast cell growth conditions were described elsewhere [23]. For FACS sorting, CRP was labelled with dye ATTO 647N (ATTO-Tec). To this end, 3.5 µL of a 2.4 mM solution of ATTO647N NHS-ester was added to 0.33 mg of CRP diluted in 110 µL of PBS buffer. The reaction mixture was stored on ice for one hour. Then, 10 µL of a 2M Tris-HCl pH 8.0 solution was added to allow an excess ATTO647N reaction, and such a fraction was removed by filtration through a Zeba Spin Desalting Column (Thermo Scientific). Induced cells were successively double-stained with a CRP-ATTO647N conjugate used at a micromolar concentration and, to quantify cell surface display, with a biotinylated anti-c-myc antibody in combination with R-phycoerythrin conjugated streptavidin (SAPC). Cells displaying double fluorescence were sorted out, propagated as described [23] and sorted again. For 18 clones, CRP binding was confirmed using individual flow cytometry and the corresponding plasmid DNA was prepared and sent out for sequencing.

### 2.4. VHH Small- and Large-Scale Purification 

Nanobody unique sequences were sub-cloned into a bacterial pET14-derived expression vector that enabled their expression as fusions of the VHH moiety to 6xHis and to a second tag, either mouse Fc or SpyTag [13,24]. Such constructs were transformed into BL21 (DE3) SOX cells for cytoplasmic expression [25] and first purified at a small scale using magnetic beads to verify the presence of the nanobody constructs in the soluble fraction, successively at a large scale using metal affinity chromatography, as reported in detail previously [24]. After analysis using SDS-PAGE, soluble protein was quantified using the Bradford method and used for assessing the binding capacity to CRP. 

### 2.5. Reconstitution of the SpyTag-SpyCatcher Complex

The fusion protein SpyCatcher002-mClover3-HisTag was produced in BL21 (DE3) bacteria using a modified pET14 vector (Supplementary Materials Figure S1). SpyTagged nanobodies and the fluorescent SpyCatcher were incubated for 1 h at 25 °C in a 4:1 molar ratio to allow for the formation of the isopeptide bonds between the two moieties of the Spy split system [26]. The reconstituted complex was evaluated by SDS-PAGE.

### 2.6. ELISA 

Nanobody specific binding to CRP was evaluated using ELISA. One hundred microliters of CRP (5 µg/mL) in PBS plus 2% BSA was used to coat the microplate wells (0.5 µg per well). Two alternative protocols were compared. In the first case, CRP was incubated overnight at 4 °C, whereas in the second, the incubation was 2 h at 37 °C. Wells were washed three times with PBST before adding purified anti-CRP nanobodies used at either 0.5 µg per well (A6, A12, B9 and H7) or 0.1 µg per well (E12) in 2% BSA in PBS buffer. After a 2-h incubation at room temperature, nanobodies were detected with mouse anti-His monoclonals (1:1000 of the commercial stock solution, Invitrogen ref. 37–2900) and visualized by means of anti-mouse HRP (1:1000 of the commercial stock solution, Sigma ref. A9044-2M) and TMB. Controls were CRP or BSA with the secondary anti-mouse HRP and CRP or BSA with both primary (anti-His) and secondary commercial antibodies. Experiments were performed in triplicate using two independent nanobody preparations and an irrelevant nanobody was used as a negative control. 

The dose-dependent detection of the antigen was evaluated using the short ELISA protocol described above by using variable concentrations of CRP (1 to 10 µg/mL) and 25 µg/mL of E12 fused to mouse Fc (E12-mFc) in combination with anti-mouse secondary antibodies fused to HRP. The experiment was performed in triplicate.

### 2.7. Immunoprecipitation Assay (IP)

IP was performed, coating 25-microliter epoxy magnetic beads (Dynabeads, Invitrogen) with 10 µg of SpyCatcher according to the manufacturer’s instructions. After washing the beads thrice in 500 μL of PBST, the SpyTagged-anti-CRP nanobodies (5 µg) and CRP (5 µg) were incubated for 2 h at 37 °C under gentle agitation. The immunoprecipitated beads were washed thrice with PBST, boiled in SDS loading buffer for 5 min at 90 °C followed by SDS-PAGE analysis.

### 2.8. Bio-Layer Interferometry (BLI) Measurement

The dissociation constant (KD) of the nanobodies for their antigen CRP was measured using BLI using an Octet K2 instrument and aminopropylsilane (APS) biosensor tips (ForteBio). The assays were performed in black 96-well plates (Greiner Bio-One), containing 200 μL per well of buffer/samples. All experiments were performed at 30 °C with shaking at 1000 rpm. CRP was used at a concentration of 25 nM. The APS tips were pre-treated in 200 μL of PBS (BioShop Canada Inc.) for 10 min, followed by equilibration in PBS for 100 s. CRP resuspended in PBS was incubated with APS tips for 900 s and unbound sites were blocked with 1% BSA (Sigma)/PBS. A baseline was obtained by incubation in PBS for 900 s, the association of anti-CRP nanobodies (concentration ranged between 10 and 1200 nM, in 0.1% BSA/PBS) was performed for 600 s followed by dissociation (600 s) in 0.1%BSA/PBS. Reference samples contained 0.1% BSA/PBS buffer. 

The obtained data were analyzed using the Octet Data Analysis HT Software (Version 11, ForteBio) after reference subtraction. Curves were aligned on the Y-axis and to the beginning of the dissociation step. The Savitzky–Golay filter was used to remove high-frequency noises from the data. Kinetic analysis was performed using a 1:1 binding model and global fitting. Kinetic parameters were considered acceptable when the coefficient correlation requirement was >80% and the chi-squared requirement was <3.0. 

### 2.9. CRP Detection by Means of An Electrochemical Impedance Biosensor

The biosensor was built by introducing some modifications to a previously described protocol [13] and using the E12 anti-CRP nanobody fused to SpyTag as the specific immunocapture component in combination with SpyCatcher previously attached to the detection surface. Electrochemical measurements were carried out using a potentiostat (EDAQ SP1) with a conventional three-electrode system composed of a gold electrode (diameter = 2 mm) as the working electrode, a platinum wire counter electrode and a Hg/Hg_2_SO_4_ (saturated with K_2_SO_4_) reference electrode.

The gold electrodes were polished manually using alpha-alumina (0.05 μm) slurry and fabricated by immobilizing 2.5 µg/mL E12 via gold-thiol chemistry. Electrochemical Impedance Spectroscopy (EIS) was carried out at open circuit potential with an amplitude of 10 mV and frequency range of 0.1–1.0 × 10^5^Hz. Nyquist plots of EIS data were fitted with the equivalent circuit using ZView software. Cyclic voltammetry (CV) was conducted at a scan rate of 20 to 200 mV s^−1^ to characterize the response of the bare electrode. All electrochemical measurements were performed in triplicate at room temperature in a 0.1 M KNO_3_ solution containing 5 mM equimolar K_3_[Fe(CN)^6^]/K_4_[Fe(CN)^6^] at a potential range of −0.7 to +0.3 V and scan rate of 20 mV s^−1^. Negligible differences were detected among repeats.

## 3. Results and Discussion

We used a llama pre-immune phage display library [14] for isolating nanobodies that are able to bind to CRP. The efficiency of the phage display library panning was evaluated after two rounds of selection by assessing the binding specificity of 90 arbitrary selected clones. The phage ELISA experiment compared the signal intensity in the presence of either the coating molecule alone (BSA, negative control) or BSA plus the target antigen CRP. The clones were considered positive when the ratio between the specific (CRP) and the control (BSA) signals, performed in triplicate experiments with the phages present in the culture supernatant, was above five. Applying such filter conditions, 36/90 clones resulted positive and were submitted for sequencing, which provided 20 correct and complete sequences, corresponding to 14 unique sequences; A6 was repeated twice and A12 six times. Three clones (A12, B9, C10) had specific-to-background ELISA scores close or higher than 15, four others (A6, A10, C1, E12) had scores of 12–13 and all had a very basic isoelectric point (Table 1). 

The yields of purified proteins and their corresponding affinities are reported together with the data relative to MW and pI. Clones A12 and H7 were also identified by yeast display.

**Figure 1:**
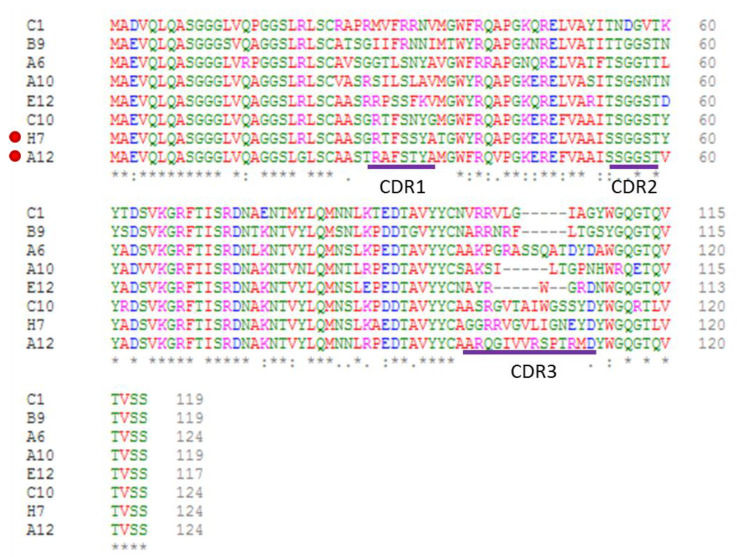
Sequence alignment of the clones with the best binding scores for CRP.

Both framework and CDR2 residues were highly conserved, whereas the CDR3 regions were, as expected for nanobodies, very heterogeneous with had no evident consensus sequence and length varying between 7 and 14 amino acids (Figure 1). The CDR1 and flanking amino acids were highly variable as well. Affinity was calculated using BLI. 

The unique sequences corresponding to the most promising clones evaluated using ELISA are reported. The sequences of clones identified by both phage and yeast display are marked with a red dot. The CDR regions are underlined, and the sequence identity, conservative mutations and other mutations are annotated on the bottom line.

The most promising clones were analyzed with the Paratome online tool [27] that should identify the residues (Antigen Binding Regions—ABRs) putatively involved in the antigen recognition more accurately than conventional methods for CDR sequence determination (Supplementary Materials Figure S2). For all the clones, the calculated ABR1 included more residues than usually attributed to standard CDR1. In the absence of structural information, it is impossible to evaluate how precise the prediction can be, but the ABR1 sequences, indeed, comprise the actual length of the variable region centered on nanobody CDR1, as evidenced by the multiple alignment analysis (Figure 1). 

In parallel, the total phagemid DNA corresponding to the output of the first round of panning of the phage display library was isolated and recombined into a vector suitable to prepare a yeast display library. Its clones were sorted by flow-cytometry using ATTO647-labeled CRP (Supplementary Materials Figure S3) and 18 single colonies were sequenced. Among these, 14 corresponded to the clone A12 and four to the clone H7 isolated after the second panning round of the phage display library (Figure 1). Some clones differed for a few point mutations of the framework residues, a condition that could reflect the individual variability among the 20 animals used to prepare the original naive library. The results urged the rescue of the clone H7 that was also isolated by phage display but not considered for the downstream characterization of the clones because of its apparent low binding capacity in the ELISA test (Table 1). It must be underlined that such a screening ELISA is made with fixed volumes of culture supernatant that are not normalized for their protein quantity and this method is prone to disadvantage clones with low productivity, independently of their binding strength. In contrast, during the yeast display selection, the actual number of binders present at the cell surface is quantified by independently staining the myc-tag fused to the nanobodies. Consequently, this method has the advantage that signals can be normalized before being gated and sorted using a flow cytometer.

In the specific case of H7, its isolation by means of two independent methods that apply alternative screening conditions strongly suggested that it was a promising binder. Finally, to evaluate the effectiveness of the isolated clones, we sub-cloned the sequences of all of the three best ELISA candidates (A12, B9, C10), with two belonging to the intermediate group (A6, E12) and one (H7) belonging to the worst group, into a vector for the expression of fusion constructs formed by the nanobodies and double 6xHis-SpyTag. Next, a small-scale analytical production was performed. The clone stability evaluation performed by ProtParam was not predictive of the actual nanobody yields reported in Table 1. Apart from C10, all the others showed an expression of soluble constructs and, therefore, underwent large-scale purification (Supplementary Materials S4). The clones yielded between 5 and 11 mg nanobody/liter of culture (Table 1). A quantitative ELISA for CRP detection was performed using the purified nanobodies and two alternative protocols. In the first case, CRP was allowed to adhere to the plates for 2 h, in the second overnight period. The data (Table 2) confirmed the nanobody specificity for their antigen. The strongest signals were obtained with B9 and E12, and the clones A12 and H7 showed a very similar, intermediate, binding capacity, whereas A6 was the less efficient binder. The results indicated clone preferences for one or the other of the CRP-coating protocols, suggesting that ad hoc optimization of the protocol can be necessary (Table 2). No CRP binding was detected when an irrelevant nanobody specific for the Her2 ectodomain was used as a negative control. 

A quantitative ELISA (mean values and corresponding standard deviations) was performed with defined amounts of purified nanobodies using two coating protocols (see M&M for experimental details)

The affinity of the selected nanobodies for CRP was quantified more precisely using BLI. The data confirmed that E12, with a KD value in the low nM range (13 nM, with an error of 3.05 × 10^−10^, Supplementary Materials Figure S5), had the highest affinity for its antigen (Table 1). A6 performed better than A12 and H7, whereas it was not possible to obtain conclusive data relative to B9 affinity (Table 1). These results suggest that the two display systems share a positive bias toward the clone A12, which was most frequently isolated in both selection procedures, despite its apparent intermediate binding strength. In contrast, whereas the phage display enriched the A6 population, the yeast display favored the accumulation of H7. Our data set is numerically very limited to draw solid conclusions but confirms previous results [15,16], according to which, library selections are not solely guided by the binder affinity but also by amplification/secretion/display parameters that can differ in each display system.

**Figure 2:**
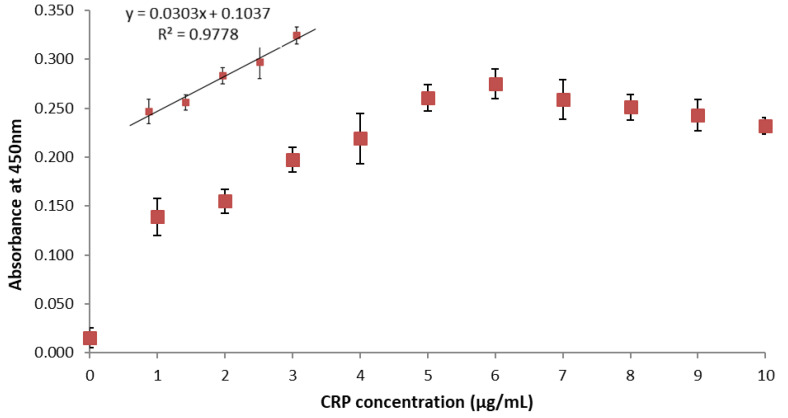
CRP detection using the reconstituted Fc-E12 construct.

The recombinant antibody fragments of such nanobodies have the advantage that they can be easily engineered using standard molecular biology techniques and subcloned to obtain reagents with different characteristics, chosen according to the requirements of the final application. To amplify the signal, E12 was produced fused to the mouse Fc domain to reconstitute IgG-like molecules suitable for ELISA in combination with HRP-functionalized anti-Fc secondary antibodies. These reagents allowed for the evaluation of the dose-dependent response to increasing amounts of CRP. As shown in Figure 2, the signal was almost linear in the range between 1 and 6 μg/mL, whereas it became saturated at higher CRP concentrations. Therefore, an ELISA test performed with this reagent seems suitable for measuring the CRP values considered to predict high-risk coronary events [7].

The dose–response curve relates the signal intensity to increasing concentrations of CRP. The antigen was detected by means of E12 fused to mouse Fc in combination with anti-mouse HRP and TMB. The data are the means of three independent experiments, and the bars indicate the standard deviation

**Figure 3:**
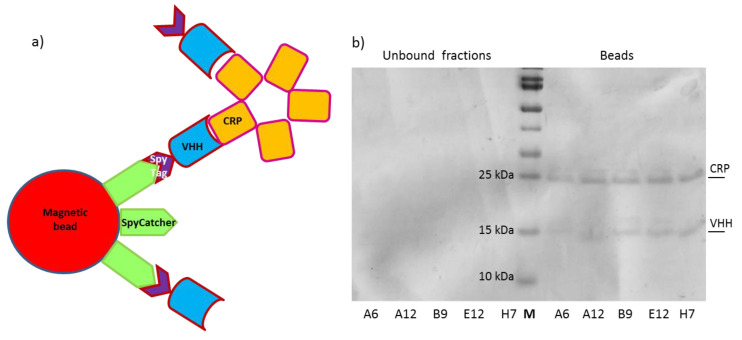
Nanobody-dependent CRP Immunoprecipitation. (**a**) Schematic representation of the procedure: nanobodies (VHHs) interact with CRP in solution and then are covalently bound to SpyCatcher-functionalized magnetic beads by means of their SpyTag. Beads are recovered with a magnet, boiled and the resulting samples separated on an SDS-PAGE (**b**). Nanobodies covalently bound to the beads do not enter the gel, but nanobodies bound only to CRP subunits are released under denaturation conditions and migrate to the expected place.

The nanobodies fused to SpyTag represent a convenient tool for functionalizing surfaces pre-immobilized with SpyCatcher [26]. This 1.1 binding strategy is rapid, highly efficient (>95% of covalent bond reconstitution) and enables the controlled directionality of the macromolecules [28,29]. The approach was successfully applied to functionalize a biosensor with nanobodies against toxic microalgae [13] and we wished to assess the possibility to use it for the quantification of low CRP concentrations using an electrochemical impedance-based biosensor. First, we successfully evaluated the reaction efficiency by incubating SpyTagged nanobodies with SpyCatcher fused to mClover3 for producing immunofluorescent reagents (chromobodies, Supplementary Materials Figure S6) and by reconstituting the SpyTag/SpyCatcher complex in an IP experiment. The anti-CRP nanobodies were initially incubated with their antigen in solution to promote the complex formation. These were then recovered by means of the available SpyTag that reacted with SpyCatcher-activated magnetic beads, as illustrated in Figure 3a. The highly specific interaction between SpyTag and SpyCatcher enabled the selective recovery of the CRP-nanobody immune complex using a magnet. The beads were boiled and VHHs and CRP were finally separated by SDS-PAGE. The stoichiometry of the reaction is difficult to predict given the multivalent characteristics of CRP and beads, but all the five tested clones were suitable for IP, although with variable efficiency according to the signal intensity of the CRP bands detectable in the gel (Figure 3b). 

**Figure 4:**
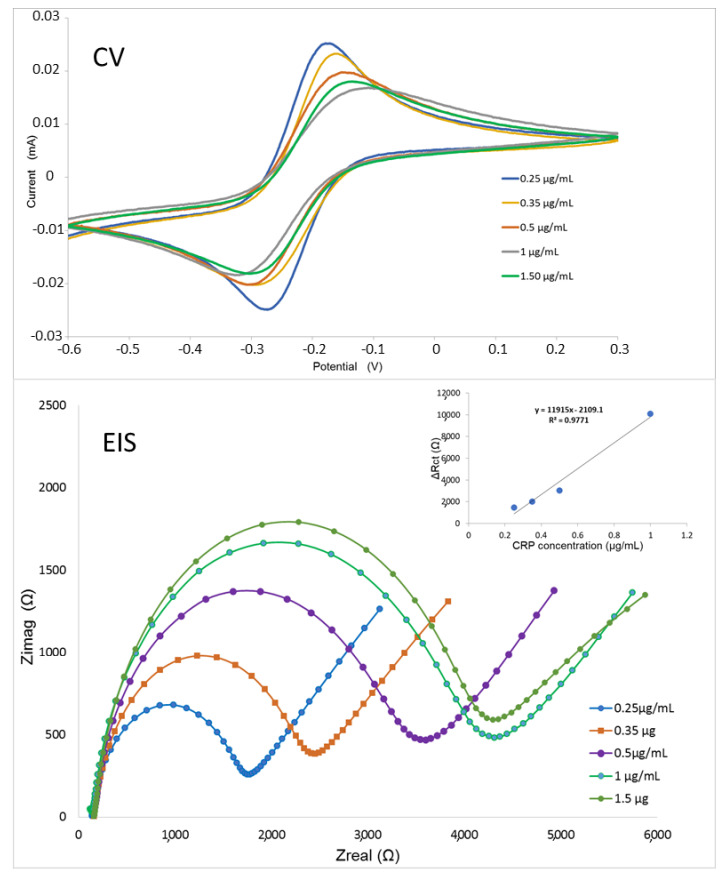
CRP detection using an electrochemical impedance biosensor.

These experiments confirmed the possibility to use our nanobodies fused to SpyTag in combination with SpyCatcher to build a stable outwards-oriented immunocapture element on the surface of an electrochemical impedance biosensor. The proof-of-concept aimed at demonstrating the feasibility of the approach rather than at providing a mature diagnostic device suitable for biological sample quantification that would require a dedicated project of optimization of both the sensor and the nanobody. The electrochemical kinetic features of the biosensor were analyzed for values ranging from 20 to 200 mV/s (Supplementary Materials Figure S7) and showed that the reaction at the electrode surface was a diffusion-controlled one. The nanobody E12 was chosen because of its excellent results in the characterization tests and used as the ligand of the electrochemical impedance spectroscopy-based biosensor. The results (Figure 4) indicate the suitability of the device, the detection limit of which was 0.21 μg/mL (Supplementary Materials Figure S8). The biosensor showed a linear range between 0.2 and 1 μg/mL of CRP and when BSA (1 μg/mL) was used as a negative control instead of CRP, only the background signal corresponding to charge transfer resistance was detected, indicating the selectivity of the biosensor (Supplementary Materials Figure S9). 

An impedance-based electric circuit was functionalized with the anti-CRP nanobody E12 and tested at different antigen concentrations. The spectra of both Cyclic Voltammetry (CV) and Electrical Impedance Spectroscopy (EIS) of a typical experiment are reported. Three repeats were performed for each antigen concentration. 

There are several already available systems for CRP quantification that exploit different diagnostic principles, require a highly variable input for both reagent and biosensor preparation and are suitable for extremely diverging concentration ranges [7,30,31]. Our biosensor is comparable with another label-free EIS prototype that exploits affirmers for capturing the antigen [32]. The two systems share the use of ligands markedly smaller than IgG and that extend roughly 3 nm away from the transducing surface. Since IgG has been already successfully used in CRP diagnostics, for instance, as a reagent in lateral flow, fluorescence immunochromatography and different sandwich ELISA assays [33,34,35,36,37] with sensitivity until 1 ng/mL^−1^, it can be expected that nanobodies might also substitute the original immunoreagents in these diagnostic methods since their small dimensions enable a higher ligand density, a condition that reduces background signal and favors sensitivity. Nanobodies are particularly stable, simple to engineer and functionalize and (as other recombinant small ligands) can be produced inexpensively and already fused to different tags suited to specific downstream applications that speed up the coating steps and, consequently, significantly reduce the analysis costs and, therefore, make diagnostics more accessible with respect to the use of IgG monoclonal antibodies. 

## 4. Conclusions

This work had the following several objectives: (i) isolating nanobodies as recombinant immunocapture reagents for affordable CRP diagnostics; (ii) comparing the efficiency of a new selection method that integrates the phage and yeast display with the standard panning protocol based on phage display only; (iii) showing the proof-of-principle that engineered recombinant nanobodies may be used for the effective immunocapture of soluble antigens on biosensor surfaces. The characterization of the clones recovered by panning confirmed that they were specific for CRP and could be produced at relatively high yields. The best candidate (E12) affinity was in the low nanomolar range and all the nanobodies were suitable for both ELISA and immunoprecipitation assays, indicating that they can bind to CRP both in solution and when immobilized on a surface. Altogether, these results confirmed the successful isolation of promising binders. Nevertheless, they must be considered as hits requiring further improvement to become lead reagents for CRP diagnostics. In silico methods have recently been shown to be highly efficient to improve the biophysical characteristics of nanobodies because their short sequence requires relatively limited computational resources for rational modeling [38,39,40,41]. We have demonstrated, with some examples, how simple nanobody engineering is in different formats suitable for different applications and expect that such anti-CRP might become a valid alternative to the conventional antibodies used thus far for CRP quantification. With respect to the already available approaches, ours, based on the use of nanobodies, may be significantly cheaper because of the lower production costs and the binder’s structural stability that enables their use for more measurement cycles and longer shelf-life [13]. This condition could enable large-scale preventive screening actions to monitor clinically relevant CRP concentrations suitable to assess vascular risk [42,43]. The sensitivity of our electrochemical device is already similar to that of sensors exploiting the same assay principles (reviewed in [30]) but we expect that further optimization of the presented EIS prototype will allow for further LOD improvements and the expansion of its linear detection range. The development of the whole pipeline, from nanobody isolation to biosensor construction and testing, is extremely rapid, technically simple and can be adapted for the quantification of any antigen. From this perspective, it represents a valuable approach for providing a rapid and reliable diagnostic response to sudden challenges, such as pandemics or massive contaminations of spread resources such as water. Of course, complex biological samples impose further challenges and require a demanding setting phase that will follow this work, which was dedicated to demonstrating the technical feasibility of the procedure.

This is the first report in which it has been investigated how nanobody selection might be affected by an integrating phage and yeast display in a unique protocol. After a common first panning round in a phage display, the successive selection and clone screening were performed in parallel using either phages or yeast cells. To obtain information about clonal diversity and to investigate whether a bias in the occurrence of clones exists, e.g., due to fast propagation in *E. coli* or yeast, micromolar target concentrations were used for yeast display screening and no efforts were made to isolate high affinity binders, e.g., by a stepwise lowering of the target concentrations in each screening round. The first observation was that the results obtained using the two protocols were partially convergent. This output was not foregone because the two selection strategies not only enable alternative screening options but use either soluble (yeast display) or surface-bound antigens (phage display). This condition might result in different structural conformations of the antigen and, in the case of CRP, in the prevalence of pentameric or monomeric forms and, consequently, in the isolation of nanobodies that might recognize different epitopes. In our specific case, the clone A12 was the most frequent in both selections (4 repeats on 20 in phages, 14 on 18 in yeast) and also the clone H7 was present in both cases. The fact that two clones were isolated by totally independent selection procedures already strongly supports the idea that these clones were specific for CRP. However, the clone panel obtained by the phage display was more diverse (14 unique sequences on 20 tested clones against 2 on 18 for the yeast selection). The two clones isolated by both protocols clearly recognize an epitope conserved in CRP molecules independently on their multimerization state. In both cases, the primary selection drive was not the nanobody affinity, since the *K*D of A12 was worse than that of E12 (isolated as a single clone only in phage display). The literature concerning the antibody fragment display efficiency is scarce, but the accurate studies performed with peptides demonstrated that some clones could overcome the concurrence during selection because of their rapidity during the amplification steps of panning [44,45]. Furthermore, the secretion efficiency of the single clones might differ in phages and yeast, determining a selection factor independent of the binder affinity for its antigen. The outcome is that the combination of such factors can represent a strong bias that questions the principle of panning as the process by which the most affine binders are promoted. It can be seen that our results do not mirror a universal condition, namely, that the number and clone characteristics obtained by the two selection strategies could vary when using other antigens. Nevertheless, we have now demonstrated that “undesirable evolution” can happen during panning. Reducing the number of panning rounds may be an opportune strategy to prevent a diversity loss that too strongly depends on uncontrolled disturbing factors. Additionally, more stringent conditions during yeast sorting might favor good but rare clones [46]. In conclusion, the combination of phage and yeast displays presented in this contribution shows experimental benefits and drawbacks that could be not anticipated by the theory. Apparently, the above-described biases and panning conditions performed at saturating antigen concentrations challenged our initial expectations of obtaining larger clone variability by screening the whole output from R1 using a yeast display and even resulted in an apparent lower clone diversity. However, the yeast display enabled signal normalization during selection, an element that renders the clone attributes comparable and the selection procedure reliable and reproducible. The reported results offer the background to conceive a further optimized panning strategy (higher stringency, competition with soluble antigen) for the phage/yeast combined selection with other antigens to integrate the collected information and learn if it is possible to synergistically exploit the potential of phage and yeast displays. Since this approach is affordable in terms of expertise, running costs and requested infrastructures, it might still represent a valid alternative to valuable binder selection methods based on deep sequencing or computational prediction [47,48] that remain inaccessible to most of the research labs.

## Figures and Tables

**Table 1:** Specific characteristics of anti-CRP nanobodies selected by the phage display protocol.

Clones	MW kDa	pI	Screening ELISA (CRP/BSA Signal)	Yield mg/L	Affinity nM
A6	13.1	9.30	11.8	9.0	51
B9	12.9	9.86	14.6	8.8	-
E12	12.8	9.51	13.3	11.1	13
C10	13.4	9.43	14.6	0	-
A12	13.2	9.34	15.5	5.3	122
H7	13.1	8.95	7.1	9.8	154
A10	12.8	9.39	12.3	-	-
C1	13.2	9.85	12.5	-	-

**Table 2:** Nanobody binding to CRP determined using an ELISA.

	Overnight Coating at 4 °C		2 h Coating at 37 °C	
	CRP	BSA	CRP	BSA
**A6**	0.476 ± 0.101	0.066 ± 0.007	0.231 ± 0.010	0.067 ± 0.005
**A12**	1.232 ± 0.420	0.070 ± 0.008	0.974 ± 0.006	0.063 ± 0.003
**B9**	1.561 ± 0.008	0.214 ± 0.014	2.284 ± 0.227	0.111 ± 0.005
**E12**	2.842 ± 0.136	0.283 ± 0.020	2.602 ± 0.450	0.202 ± 0.016
**H7**	1.468 ± 0.057	0.080 ± 0.014	0.904 ± 0.100	0.066 ± 0.002
**antiHis+ antimouse HRP**	0.070 ± 0.005	0.069 ± 0.022	0.066 ± 0.001	0.066 ± 0.005
**antimouse HRP**	0.063 ± 0.004	0.060 ± 0.013	0.062 ± 0.003	0.062 ± 0.003

## Data Availability

Data will be provided after request.

## Supplementary Materials

The following are available online at https://www.mdpi.com/article/10.3390/bios11120496/s1, Figure S1: Characteristics of the vector used for the production of the SpyCatcher002-mClover3-HisTag fusion protein, Figure S2: Results of Paratome predictions, Figure S3: Specific enrichment of yeast clones displaying anti-CRP nanobodies using flow-cytometry, Figure S4: SDS-PAGE corresponding to nanobody purifications, Figure S5: E12 affinity calculation, Figure S6: Reconstitution of the nanobody-SpyTag:SpyCatcher-mClover3 complex, Figure S7: Kinetic analysis of the electrochemical biosensor, Figure S8: Characterization of the impedance-based biosensor sensitivity, Figure S9: Negative control for the electrochemical impedance biosensor, Table S1: APS sensor with immobilised CRP (25 nM) responses, obtained for E12 nanobodies at con-centrations of (a) 10; (b) 50; (c) 100; (d) 300; (e) 600; (f) 1200 nM; (g) non-specific binding 1200 nM E12 nanobodies to the APS sensor surface. Red lines mean theoretical response after using 1:1 fitting model.
