# Supplementary material for: Native llama Nanobody Library Panning Performed by Phage and Yeast Display Provides Binders Suitable for C-Reactive Protein Detection
Source: Biosensors (Basel). 2021 Dec 3;11(12):496. doi: 10.3390/bios11120496 (PMC8699515; doi:10.3390/bios11120496)
Supplement: Supplementary file 1 [file biosensors-11-00496-s001.zip › biosensors-1459220-supplementary.pdf]

Supplementary Material

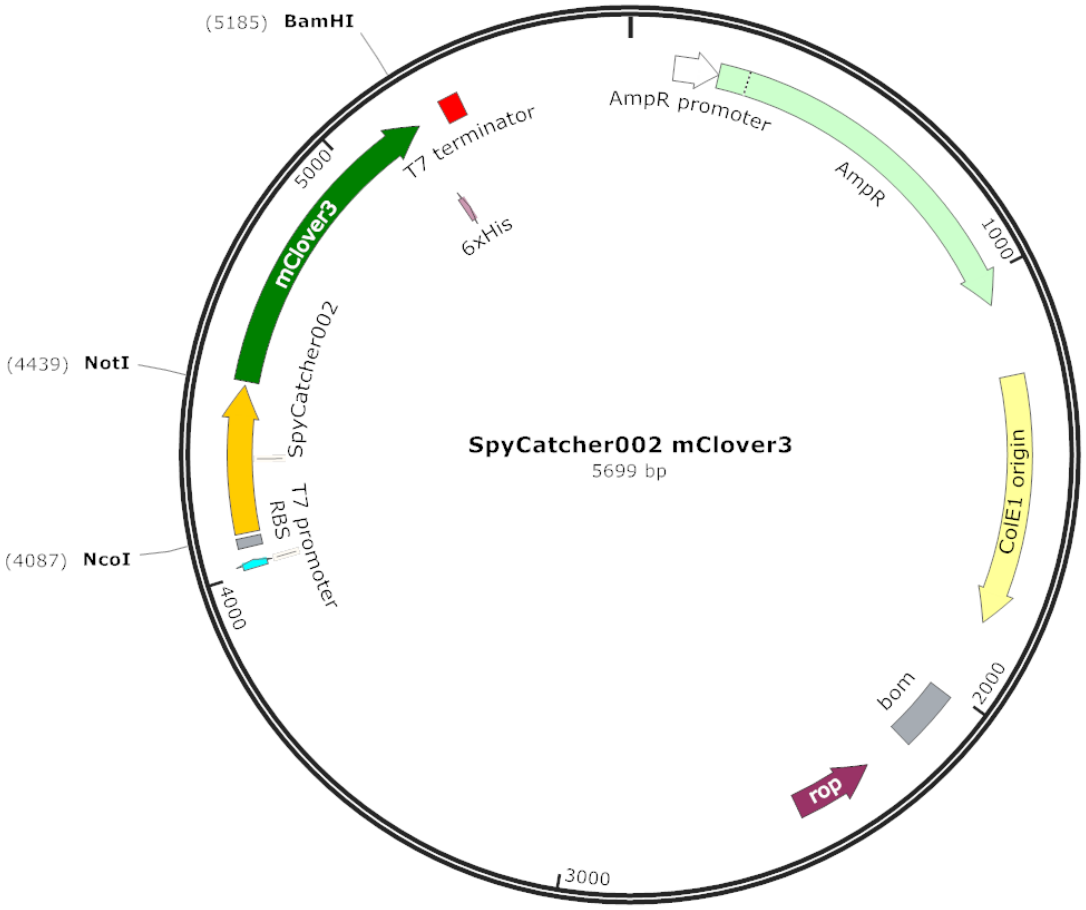

Figure S1. Characteristics of the vector used for the production of the SpyCatcher002-mClover3-HisTag fusion protein.

>A6_CRP
MAEVQLQASGGGLVRPGGSLRLSCAVSGGTLSNYAVGWFRRAPGNQRELVATFTSGGTTLYADSVKGRFTISRDNLKNTV
YLQMNSLKPEDTAVYYCAAKPGRASSQATDYDAWGQGTQVTVSS

ABR1: GTLSNYAVG (29-37) ABR2: LVATFTSGGTTLYA (49-62) ABR3: AAKPGRASSQATDYDA (98-113)
>B9_CRP
MAEVQLQASGGGSVQAGGSLRLSCATSGIIFRNNIMTWYRQAPGKNRELVATITTGGSTNYSDSVKGRFTISRDNTKNTV
YLQMSNLKPDDTGVYYCNARRNRFLTGSYGQGTQVTVSS

ABR1: IIFRNNIMT (29-37) ABR2: LVATITTGGSTNY (49-61) ABR3: ARRNRFLTGS (99-108)

>E12_CRP
MAEVQLQASGGGLVQAGGSLRLSCAASRRPSSFKVMGWYRQAPGKQRELVARITSGGSTDYADSVKGRFTISRDNAKNTV
YLQMNSLEPEDTAVYYCNAYRWGRDNWGQGTQVTVSS

ABR1: RRPSSFKVMG (28-37) ABR2: LVARITSGGSTDY (49-61) ABR3: AYRWGRDN (99-106)

>C10_CRP
MAEVQLQASGGGLVQAGGSLRLSCAASGRTFSNYGMGWFRQAPGKEREFVAAITSGGSTYYRDSVKGRFTISRDNAKNTV

YLQMNSLKPDDTAVYYCAASRGVTAIWGSSYDYWGQRTLVTVSS

ABR1: RTFSNYGMG (29-37) ABR2: FVAAITSGGSTYYR (49-62) ABR3: AASRGVTAIWGSSYDY (98-113)

>A12_CRP
MAEVQLQASGGGLVQAGGSLGLSCAASTRAFSTYAMGWFRQVPGKEREFVAAISSGGSTVYADSVKGRFTISRDNAKNTV
YLQMNNLRPEDTAVYYCAARQGIVVRSPTRMDYWGQGTQVTVSS

ABR1: TRAFSTYAMG (28-37) ABR2: FVAAISSGGSTVY (49-61) ABR3: ARQGIVVRSPTRMDY (99-113)

>H7_CRP
MAEVQLQASGGGLVQAGGSLRLSCAASGRTFSSYATGWYRQAPGKERELVAAISSGGSTYYADSVKGRFTISRDNAKNTV
YLQMNSLKAEDTAVYYCAGGRRVGVLIGNEYDYWGQGTLVTVSS

ABR1: RTFSSYATG (29-37) ABR2: LVAAISSGGSTYYA (49-62) ABR3: AGGRRVGVLIGNEYDY (98-113)

>A10_CRP
MAEVQLQASGGGLVQAGGSLRLSCVASRSILSLAVMGWYRQAPGKERELVASITSGGNTNYADVVKGRFIISRDNAKNTV
NLQMNTLRPEDTAVYYCSAKSILTGPNHWRQETQVTVSS

ABR1: RSILSLAVMG (28-37) ABR2: LVASITSGGNTNYA (49-62) ABR3: AKSILTGPNH (99-108)

>C1_CRP
MADVQLQASGGGLVQPGGSLRLSCRAPRMVFRRNVMGWFRQAPGKQRELVAYITNDGVTKYTDSVKGRFTISRDNAENTM
YLQMNNLKTEDTAVYYCNVRRVLGIAGYWGQGTQVTVSS

ABR1: PRMVFRRNVMG (27-37) ABR2: LVAYITNDGVTKYT (49-62) ABR3: NVRRVLGIAGY (98-108)

Figure S2. Results of Paratome predictions, the software predicts the Antigen Binding Regions (ABRs, reported in color) that should correspond to nanobody residues potentially involved in the paratope.

Yeast cells were labeled with CRP-ATTO647N conjugate (target binding) and biotinylated anti-c-myc antibody in combination with R-phycoerythrin conjugated streptavidin (surface presentation). Double labeled cells (the upper right population) were sorted out (round 1) and immediately resorted (round 1 resort). The twice sorted yeasts were plated for single clone analysis.

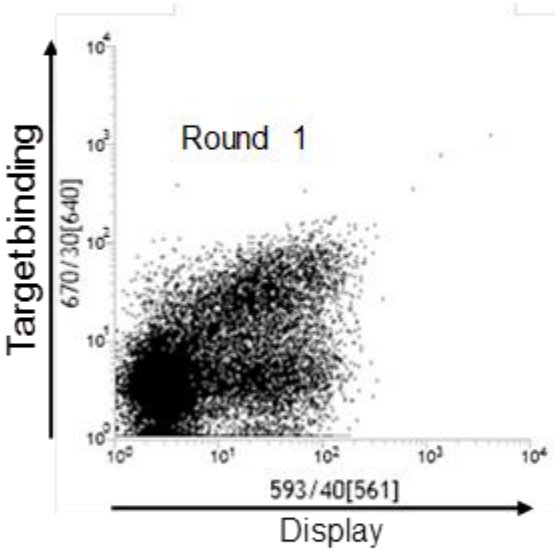

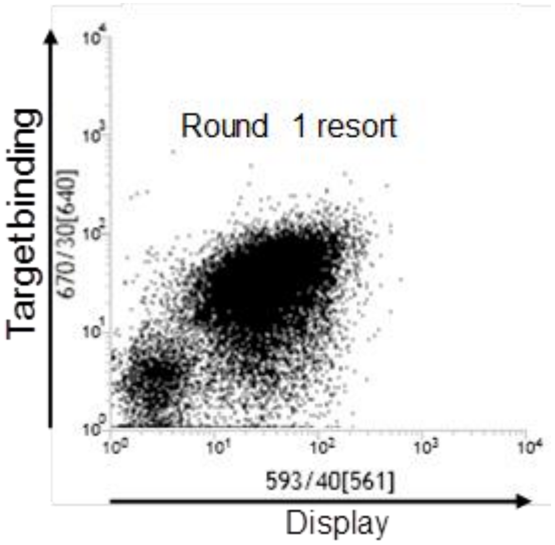

Figure S3. Specific enrichment of yeast clones displaying anti-CRP nanobodies by flow-cytometry. The whole population of yeast displayed nanobodies was analyzed and sorted by flow-cytometer.

Nanobody constructs were first purified in small-scale to verify their soluble expression and then in preparative large-scale. Only fractions showing no apparent contaminants were pooled and desalted. M: molecular weight marker; Sn: supernatant, P: pellet; Ft: flow-through; E: elution fractions; D: desalting fractions

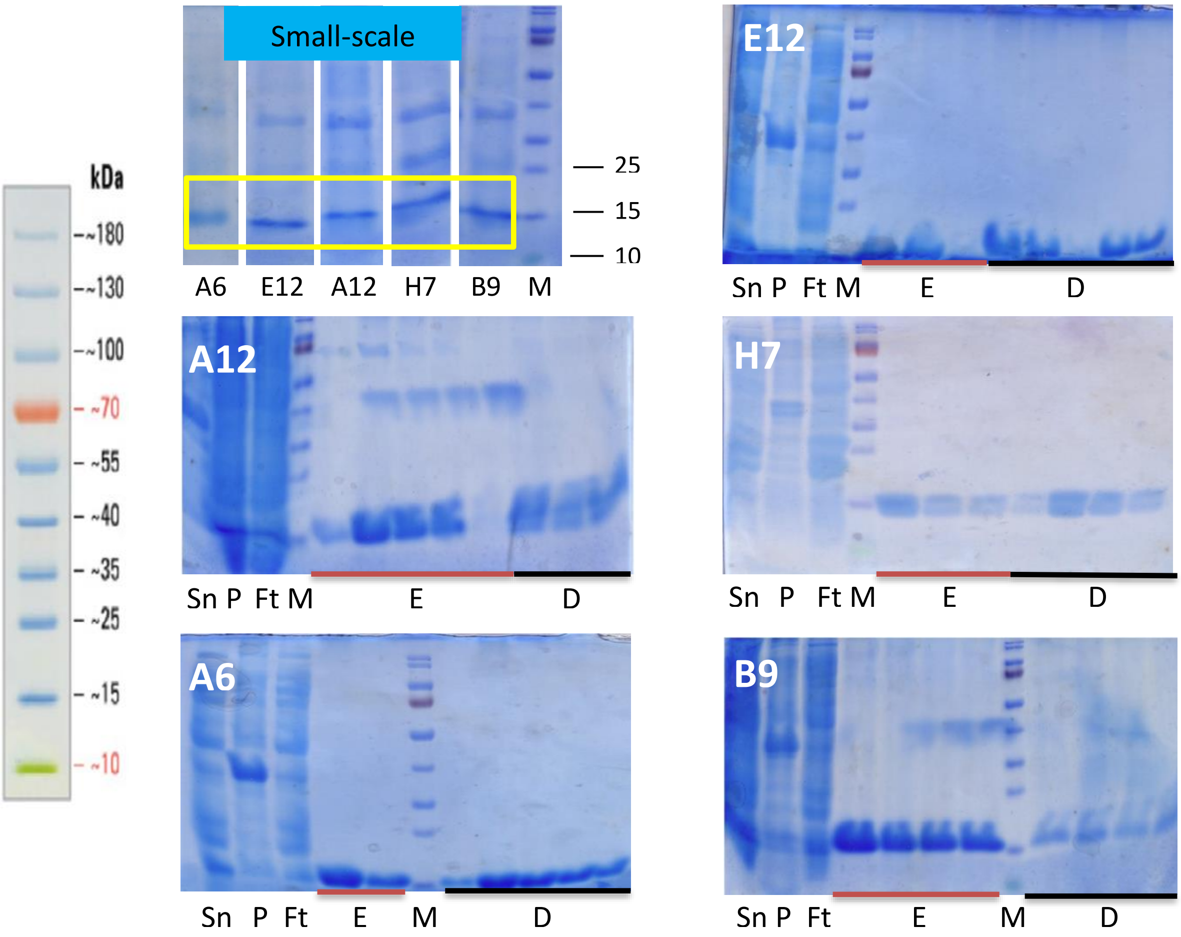

Figure S4. SDS-PAGE corresponding to nanobody purifications.

The obtained data were analyzed using the Octet Data Analysis HT Software (Version 11, ForteBio) after reference subtraction. The reference was the value obtained using the same APS sensor activated with the ligand (CRP, 25 nM) in the absence of analyte and was subtracted according to the manufacturer's recommendations. Curves were aligned on the Y-axis and to the beginning of the dissociation step, the Savitzky-Golay filtering was used to remove high-frequency noises from the data applying the Pall Fortebio LLC, Octet Data Analysis High Throughput system. The kinetic analysis was performed using a 1:1 binding model and global fitting.

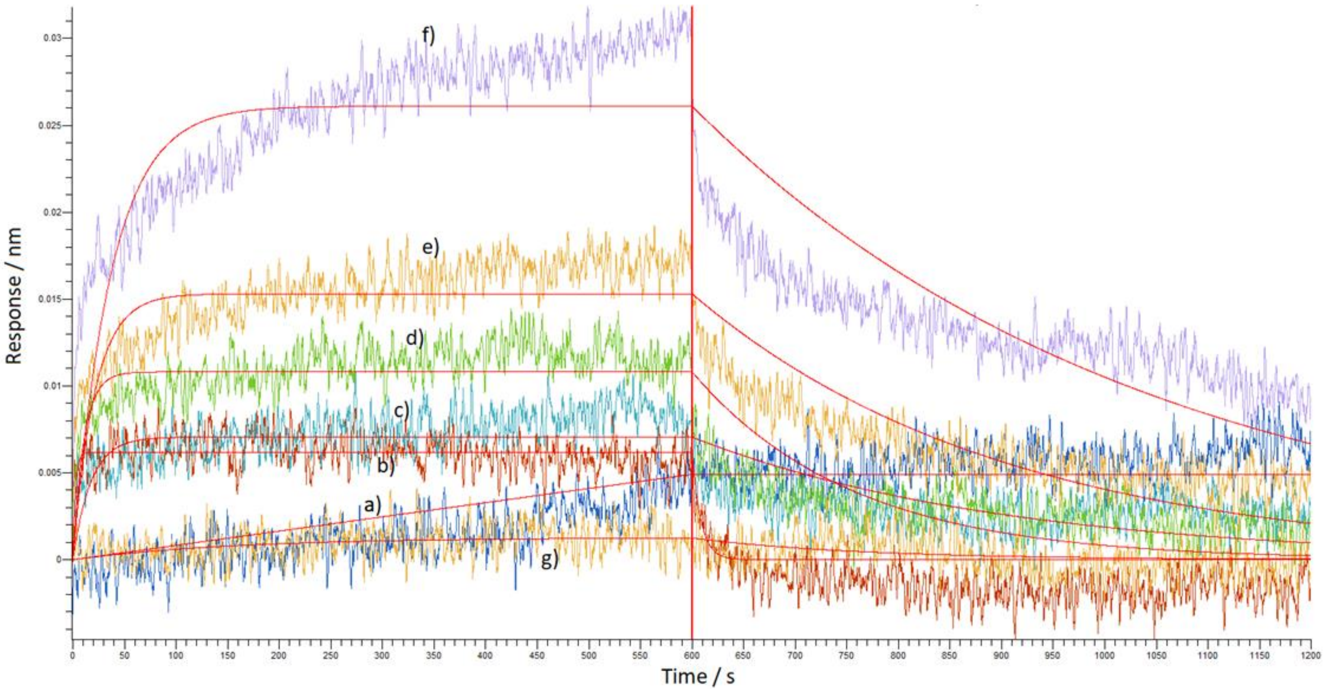

Figure S5. E12 affinity calculation.

Table S1. APS sensor with immobilised CRP (25nM) responses, obtained for E12 nanobodies at concentrations of a) 10; b) 50; c) 100; d) 300; e) 600; f) 1200 nM; g) non-specific binding 1200nM E12 nanobodies to the APS sensor surface. Red lines mean theoretical response after using 1:1 fitting model.

Receptors	K_D (M)	K_D Error	k_a (1/Ms)	k_a Error	k_{dis} (1/s)	k_{dis} Error	X^2	R^2
**Nanobodies**	1,31E-08	3,22E-10	1,96E+05	4,72E+03	2,57E-03	1,19E-05	0,2941	0,8662

K_D – Affinity constant
k_a – Rate of association
k_{dis} – Rate of dissociation

X^2 is the sum of the squared deviations, which generally should be below 3. X^2 is a measure of error between the experimental data and the fitted line. A smaller X^2 indicates a better fit.

The R2 value indicates how well the fit and the experimental data correlate.

The theoretical plots are the red lines on pictures (Fig. 1); kinetic parameters are acceptable according to the manufacturer's indications, namely the coefficient correlation r is >80% and the chi-squared r <3.0.

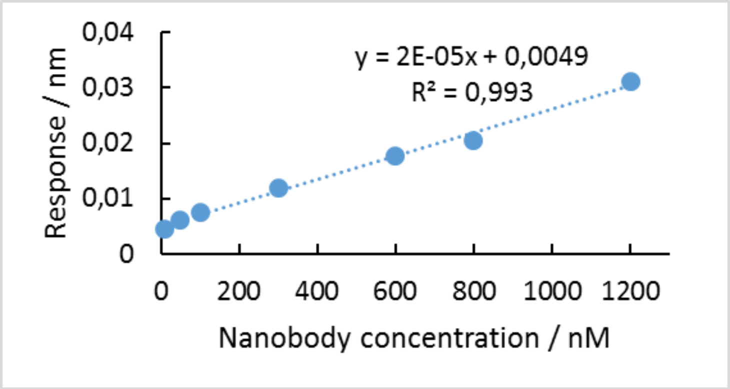

Figure S6. Reconstitution of the nanobody-SpyTag:SpyCatcher-mClover3 complex.

Purified SpyTagged nanobodies were incubated with SpyCatcher-mClover3 to promote the formation of the covalent binding between the two tag moieties. The reaction products were the separated by SDS-PAGE and the arrows indicate the protein bands corresponding to: i) the reconstituted SpyTag-SpyCatcher complex (red); the SpyCatcher-mClover3 fusion protein (green); the nanobodies (black). The fluorescent fusion protein appears as a duet.

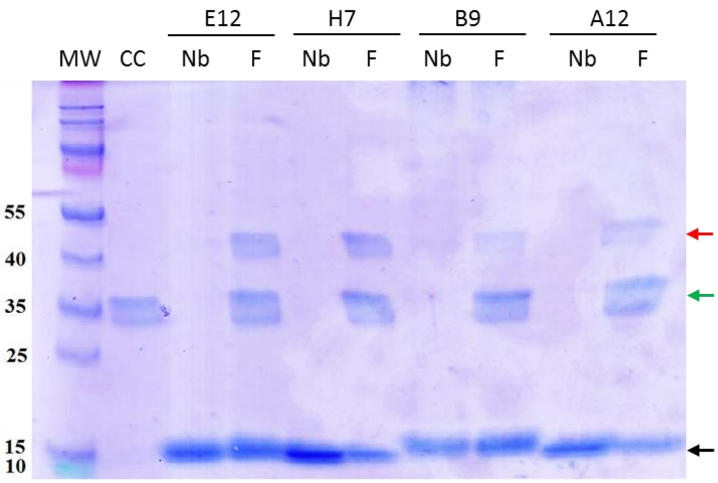

Figure S7. Biosensor electrochemical kinetic analysis.

The biosensor was analyzed for values ranging from 20 to 200 mV/s and the results demonstrated that the reaction at the electrode surface was a diffusion-controlled one

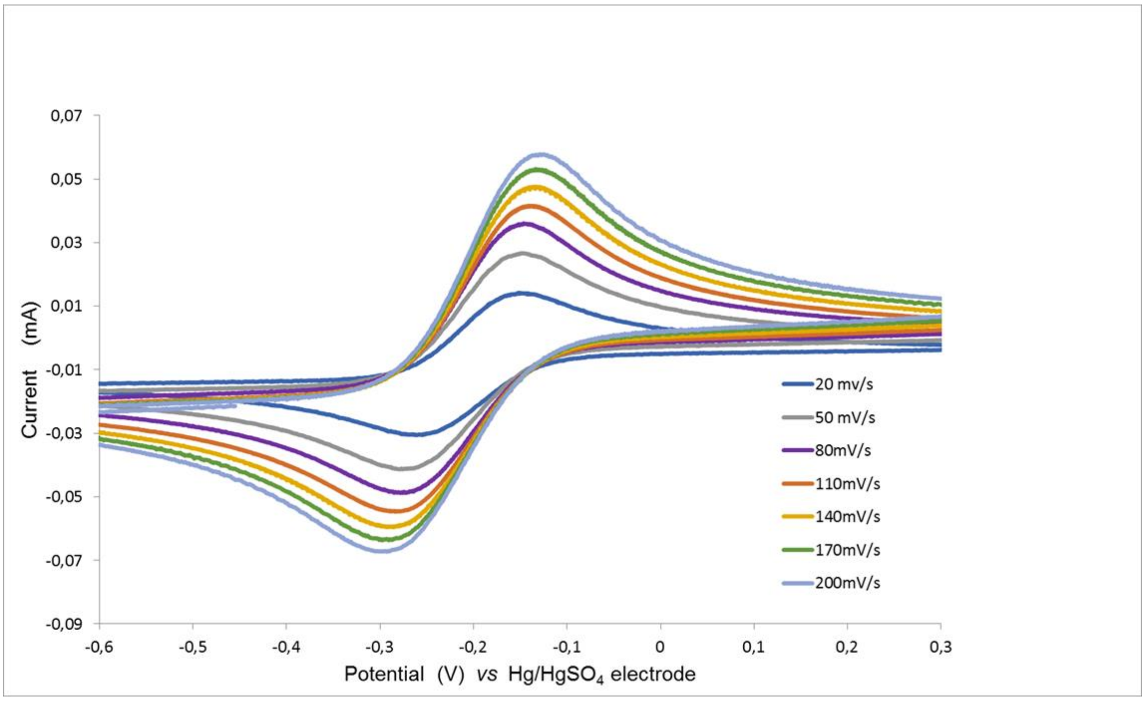

Figure S8. Characterization of the impedance-based biosensor sensitivity.

Experimental evaluation of CRP concentrations between 0.25 and 1 µg/Ml. Calculation of the limit of detection (LOD) was performed considering the experimental data:

CRP conc (µg)	Rct (ohms)
0,25	1456
0,35	2020
0,5	3023
1	10087

These were computed to obtain the values of slope and intercept standard deviation:

ANOVA

	df	*SS*	*MS*	*F*	*Significance F*
Regression	1	47206783,23	47206783,23	85,38019	0,01151
Residual	2	1105801,774	552900,8872		
Total	3	48312585			

	Coefficients	*Standard Error*	*t Stat*	*P-value*	*Lower 95%*	*Upper 95%*	*Lower 95.0%*	*Upper 95.0%*
Intercept	-2109,052632	772,367113	-2,730634948	0,112023	-5432,28	1214,174835	-5432,280099	1214,174835
X Variable 1	11915,33835	1289,519217	9,240140192	0,01151	6366,985	17463,69172	6366,984967	17463,69172

**S.D of Intercept**	**772,367113**	
**Slope**	11915,33835	
		**µg/mL**
**LOD**	**{3.3*(S.D of Intercept/Slope)}**	**0,21391**

The biosensor activated with anti-CRP nanobodies was used at the standard conditions but BSA (1 µg/mL) was used instead of CRP.

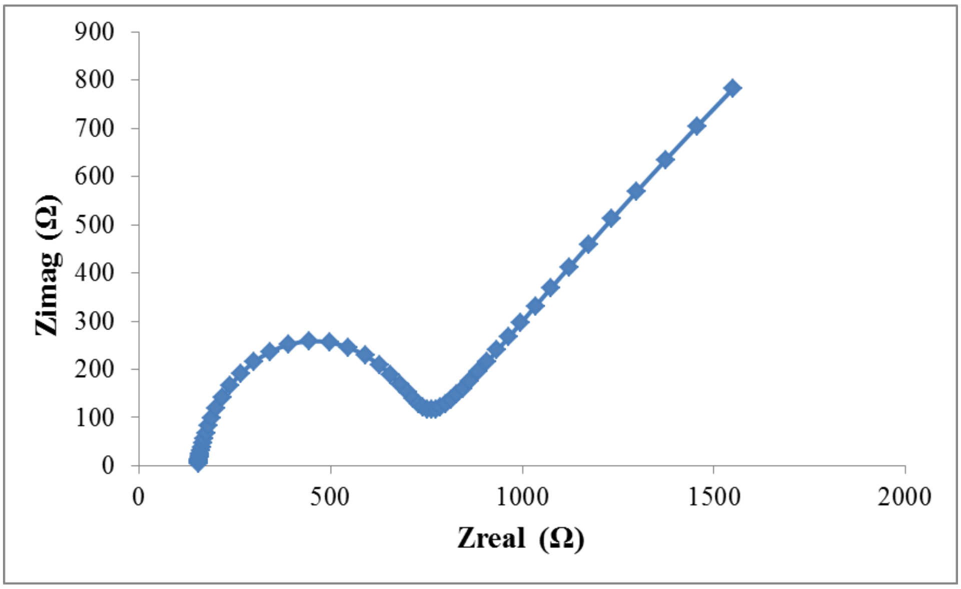

Figure S9. Negative control for the electrochemical impedance biosensor.
